# Insecticidal and Attractant Activities of *Magnolia citrata* Leaf Essential Oil against Two Major Pests from Diptera: *Aedes aegypti* (Culicidae) and *Ceratitis capitata* (Tephritidae) †

**DOI:** 10.3390/molecules26082311

**Published:** 2021-04-16

**Authors:** Ngoc Anh Luu-Dam, Nurhayat Tabanca, Alden S. Estep, Duy Hung Nguyen, Paul E. Kendra

**Affiliations:** 1 Vietnam Academy of Science and Technology (VAST), Graduate University of Science and Technology, No. 18 Hoang Quoc Viet Road, Cau Giay District, Hanoi 100803, Vietnam; ngocanh@vnmn.vast.vn (N.A.L.-D.); bochunghg@gmail.com (D.H.N.); 2 Vietnam National Museum of Nature, Vietnam Academy of Science and Technology (VAST), No.18 Hoang Quoc Viet Road, Cau Giay District, Hanoi 100803, Vietnam; 3 United States Department of Agriculture-Agricultural Research Service (USDA-ARS), Subtropical Horticulture Research Station (SHRS), 13601 Old Cutler Rd., Miami, FL 33158, USA; 4 United States Department of Agriculture-Agricultural Research Service (USDA-ARS), Center for Medical, Agricultural, and Veterinary Entomology (CMAVE), Gainesville, FL 32608, USA; alden.estep@usda.gov

**Keywords:** Magnoliaceae, mosquito, medfly, fruit fly, GC-MS, aliphatic aldehydes, citronellal, geranial, citral

## Abstract

In this study, *Magnolia citrata* Noot and Chalermglin (Magnoliaceae) essential oil (MCEO) was evaluated for insecticidal activity against the yellow fever mosquito *Aedes aegypti* and attractant activity for the Mediterranean fruit fly *Ceratitis capitata*. The leaves of *Magnolia citrata* (Giổi chanh) were collected from northwestern Vietnam, and the water-distilled MCEO was analyzed by gas-chromatography and mass spectrometry (GC-MS). The major constituents of MCEO were identified as linalool 19%, geranial 16%, citronellal 14%, neral 14%, and sabinene 12%. MCEO showed 100% mortality at 1 μg/μL against 1st instar larvae of *Ae. aegypti* (Orlando strain, ORL), and the oil exhibited 54% (ORL) and 68% (Puerto Rico strain) mortality at 5 μg/mosquito against *Ae. aegypti* adult females. Initial screens showed that MCEO had weak insecticidal activity compared to the positive control permethrin. In bioassays with sterile male *C. capitata*, MCEO exhibited moderately strong attraction, comparable to that observed with a positive control, *Tetradenia riparia* essential oil (TREO). Herein, the insecticidal and attractant activities of MCEO are reported for the first time.

## 1. Introduction

The extensive and long-term application of pesticides has resulted in an accumulation of residues in food, water, soil, and other environmental components. The negative impact of pesticides on biodiversity, and increasing pesticide resistance, have prompted more research on alternative control strategies using botanicals, biological, cultural, and other sustainable methods for integrated management of existing and invasive pests [1,2,3,4,5,6]. Plants synthesize a diverse array of molecules that can be exploited to develop novel pest control agents [7,8,9]. Vietnam is a tropical Asian country with extraordinarily rich plant biodiversity, and many of the indigenous plants are known to have medicinal properties; the country’s rich forest ecosystems provide an abundant opportunity to search for phytochemicals with potential applications for the management of major insect pest species [10,11,12]. *Aedes aegypti* L. (Diptera: Culicidae) mosquitoes are the major vector for the transmission of several uncontrolled, life-threatening viral diseases, which are more frequent in hot and humid climate regions [13,14], and thus effective mosquito control of both larval and adult stages can reduce the impact of vector-borne diseases. As an economically important agricultural pest, the Mediterranean fruit fly, *Ceratitis capitata* (Wiedemann) (Diptera: Tephritidae), is one of the most serious invasive pests in tropical and subtropical regions around the world [15]. It attacks a wide range of fruits and vegetables, with larval infestation causing severe damage to many commercially important tropical and subtropical crops [16,17]. Thus, identification of effective attractants for early detection and suppression of *C. capitata* populations is critical for managing this pest and reducing crop losses.

**Figure 1:**
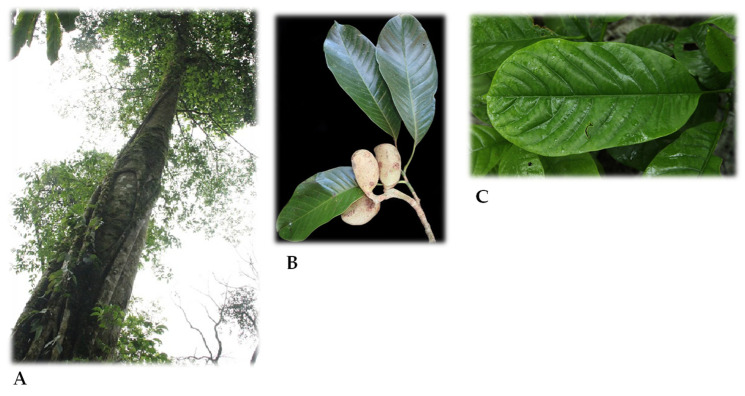
(**A**) General view of *Magnolia citrata* Noot and Chalermglin; (**B**): leaves with young fruit; (**C**) mature leaves of *M. citrata*. Photos courtesy of Mrs. Tu Bao Ngan and Mr. Bui Văn Hương. Photos provided by N.A.L-D.

The genus *Magnolia* L. (Magnoliaceae) is one of the oldest families of flowering plants [18]. The genus comprises about 245 taxa occurring in temperate and tropical areas of Southeast and East Asia, North America, the Antilles, and Central and South America [19]. *Magnolia* species have attracted a great deal of research interest due to the presence of biphenolic phytochemicals magnolol and honokiol, which possess diverse pharmacological properties, including inhibition of the central nervous system, prevention of cardiovascular disease, anti-inflammatory, antimicrobial, antioxidative, and free radical scavenging activity [20,21,22]. *Magnolia* extracts and their bioactive chemicals have been evaluated for their promising insecticidal activity. A lignan, epimagnoline A, was isolated from flower buds of *M. fargesii* (Finet and Gagnep.) [23]. Larvicidal activity of different solvent extracts of *M. salicifolia* Maxim. showed good to moderate effect on fourth instar larvae of *Aedes aegypti* [24]. Essential oils from *M. grandiflora* L. were tested for insecticidal and biting deterrent activity against *Ae. aegypti,* and the seed oil exhibited biting deterrence similar to the standard insect repellent DEET (*N*,*N*-diethyl-3-methylbenzamide) [25]. Tests for insecticidal susceptibility to ethanol extracts from *M*. *dealbata* Zucc. [26] and *M. schiedeana* Schltl. were carried out against the tephritid fruit fly *Anastrepha ludens* (Loew) [27]. Sarcotesta extract from *M*. *dealbata* [26], as well as the seed and sarcotesta extracts from *M. schiedeana,* showed potential insecticidal activity against adults of *A. ludens* [27]. *Magnolia citrata* Noot and Chalermglin is an evergreen tree (Figure 1) that grows naturally in three localities in Thailand and at one locality in Vietnam [28,29,30]. Leaves are locally known as Giổi chanh’ due to strong citrus or lemongrass odor and used as spices in traditional food preparations by the people of Ha Giang [31]. In a search for biorational insecticidal agents from natural sources that could be useful in future pest management strategies, we (i) isolated *M. citrata* essential oil (MCEO) from leaves, (ii) identified the chemical content of MCEO, (iii) evaluated MCEO for potential insecticidal activity against larval and adult *Ae. aegypti*, and (iv) assayed MCEO for potential attraction of sterile male *C. capitata*. This study represents the first investigation of MCEO for insecticidal and attractant properties.

## 2. Results and Discussion

The gas chromatography-mass spectrometry (GC-MS) analysis of MCEO using a nonpolar column revealed the presence of 41 chemical components with a composition of 98% (Table 1). The major compounds of MCEO were identified as linalool 19.1%, geranial 15.7%, citronellal 14.1%, neral 13.5%, and sabinene 12.4%. The MCEO was dominated by oxygenated monoterpenoids (77.8%), followed by monoterpene hydrocarbons (17.3%), sesquiterpene hydrocarbons (0.8%), oxygenated sesquiterpenes (0.3%), and others (1.8%).

*Magnolia citrata* is listed under the International Union for Conservation of Nature (IUCN) Red List criteria as Least Concern (LC) category [30]. To avoid extinction and to conserve *M. citrata* in Vietnam, MCEO composition has been poorly explored despite its distinctive odor [31]. The existing literature showed that MCEO was rich in linalool, citronellal, and citral isomers (neral and geranial) [32], which corresponds with our results. 

**Table 1:** Chemical composition of *Magnolia citrata* oil (MCEO).

RI ^a^	RI ^b^	Compounds	%	Identification Method ^a,c,d^
851	850	(*Z*)-3-hexen-1-ol	0.6	MS, RI, std
920	924	α-thujene	0.4	MS, RI
926	932	α-pinene	0.1	MS, RI, std
968	969	sabinene	12.4	MS, RI, std
971	974	β-pinene	0.5	MS, RI, std
981	981	6-methyl-5-hepten-2-one	1.2	MS, RI, std
985	988	myrcene	0.4	MS, RI, std
1020	1020	*p*-cymene	0.8	MS, RI, std
1024	1024	limonene	0.4	MS, RI, std
1026	1026	1,8-cineole	0.3	MS, RI, std
1032	1032	(*Z*)-β-ocimene	1.2	MS, RI, std
1043	1044	(*E*)-β-ocimene	0.1	MS, RI, std
1053	1054	γ-terpinene	0.1	MS, RI, std
1064	1065	*cis*-sabinene hydrate	0.9	MS, RI
1082	1067	*cis*-linalool oxide (furanoid)	0.3	MS, RI, std
1101	1095	linalool	19.1	MS, RI, std
1107	1118	*cis*-*p*-menth-2-en-1-ol	0.1	MS, RI, std
1120	1136	*trans*-*p*-menth-2-en-1-ol	0.1	MS, RI, std
1137	1144	neo-isopulegol	0.1	MS, RI
1142	1145	isopulegol	0.8	MS, RI
1150	1148	citronellal	14.1	MS, RI
1157	1167	neoiso-isopulegol	0.1	MS, RI
1165	1173	rosefuran epoxide	0.1	MS, RI
1174	1174	terpinen-4-ol	1.6	MS, RI, std
1190	1186	α-terpineol	0.2	MS, RI, std
1220	1227	nerol	0.4	MS, RI
1226	1223	citronellol	6.5	MS, RI, std
1235	1235	neral	13.5	MS, RI
1245	1249	piperitone	0.1	MS, RI, std
1252	1257	methyl citronellate	0.9	MS, RI
1265	1264	geranial	15.7	MS, RI
1268	1271	citronellyl formate	0.4	MS, RI
1323	1312	citronellic acid	3.4	MS, RI
1405	1417	β-caryophyllene	0.1	MS, RI, std
1439	1452	α-humulene	tr	MS, RI, std
1443	1458	alloaromadendrene	0.2	MS, RI, std
1472	1489	β-selinene	0.4	MS, RI
1480	1498	α-selinene	0.1	MS, RI
1562	1582	caryophyllene oxide	0.3	MS, RI, std
1584	1602	ledol	tr	MS, RI
1589	1608	humulene epoxide II	tr	MS, RI
		Total	98.0	

^a^ RI: retention indices calculated on DB-5MS; ^b^ RI_lit_: retention indices from Adams Library [33]; tr: trace < 0.1; ^c^ MS: identified on the basis of computer matching of the mass spectra with those of the Adams Library [33], NIST [34], Wiley [35], MassFinder [36], FFNSC 3 libraries [37]; ^d^ std: authentic compounds on the DB-5MS column.

Initial larval activity testing MCEO against 1st instar *Ae. aegypti* (Orlando, ORL strain, insecticide-susceptible) showed 100%, 40%, 20%, and 0% mortality at the concentration of 1.0, 0.5, 0.25, and 0.1 μg/μL, respectively, while positive control permethrin had 100% mortality at 31 pg/μL and solvent control (DMSO) showed 0% mortality. In adult topical bioassays, MCEO was tested against female *Ae. aegypti* (ORL and Puerto Rico (PR) strain (pyrethroid-resistant)) and the oil demonstrated 53.89% (±23.35) and 67.65% (±17.74) mortality at 5 μg/mosquito, respectively. Permethrin showed 60% (±20) mortality of the ORL and 10% (±10) mortality against the PR strain at 0.69 ng/μL. Control mortality of solvent (acetone) was 0%.

MCEO demonstrated 100% mortality at 1 μg/μL (1000 ppm) against 1st instar larvae while the activity dropped quickly at lower concentrations. Previous studies of larvicidal activity of dominant compounds in MCEO were investigated against *Ae. aegypti* larvae, and they exhibited good to moderate activity. For example, the larvicidal activity of citral, which contains geranial and neral, showed 100% mortality at 100 ppm in 24 h against 4th instar *Ae. aegypti* [24]. Pandey et al. [38] reported that linalool displayed 100% mortality at 200 ppm in 24 h post-treatment against 4th instar larvae of *Ae. aegypti*. Waliwitiya et al. [39] have also reported that citronellal exhibited larvicidal activity against all four larval stages of *Ae. aegypti* (LC_50_ values 10.3–40.8 mg/L). Cheng et al. [40] found that sabinene had larvicidal activity at LC_50_ of 74.1 mg/mL against fourth-instar larvae after 24 h treatment. MCEO is not considered suitable for further evaluation due to its weak activity in both assays, which is below our laboratory thresholds of required larvicidal activity greater than 80% at 0.1 μg/μL and/or >80% mortality in adult topical assays. However, plant extracts are often considered less harmful to the environment and are perceived as more natural. These natural products represent possible alternatives to current control methods. Previous studies by our research group revealed that the PR strain of *Ae. aegypti* was highly resistant to permethrin compared with the ORL strain in topical adult assays [41,42]. Norris et al. [43] highlighted that plant essential oils could complement or synergize pyrethroids. Future work with combinations of pyrethroids and plant essential oils needs to be done to determine the viability of use in integrated pest management strategies. Therefore, plant oils might offer potential alternatives to reduce resistance in mosquito populations.

**Figure 2:**
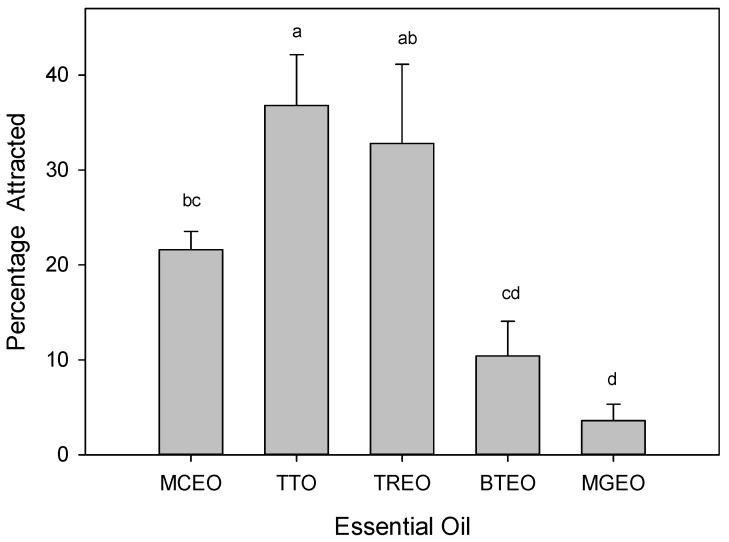
Attraction (mean ± SE) of male Mediterranean fruit fly, *Ceratitis capitata*, to five essential oils presented in short-range attraction bioassays. Response observed with *Magnolia citrata* essential oil (MCEO) was compared with response to two known strong attractants, tea tree oil (TTO) [44], and *Tetradenia riparia* essential oil (TREO) [46], and two mild attractants, blue tansy essential oil (BTEO) [47] and mastic gum essential oil (MGEO) [45]. Bars topped with the same letter are not significantly different (Tukey HSD mean separation, *p* < 0.05).

In short-range attraction bioassays with male *C. capitata*, there were significant differences in behavioral response to the five essential oil treatments (*F* = 8.509; df = 4,20; *p* < 0.001; Figure 2). As seen in previous studies [44,45,46,47], highest attraction was observed with TTO (tea tree oil) [44]. Attraction to MCEO was comparable to that observed with TREO (*Tetradenia riparia* essential oil), a strong attractant [46], but also comparable to that observed with BTEO (blue tansy essential oil), a milder attractant [47]. However, attraction to MCEO was significantly greater than that observed with MGEO (mastic gum essential oil), a weak attractant [45], and significantly less than that observed with TTO, the best essential oil attractant identified to date [44,45,46,47]. The combined results indicate that MCEO is a moderately strong attractant for male *C. capitata*.

Previous chemical analyses indicated that the major components in the highly attractive TTO consisted of terpinen-4-ol (41.8%), γ-terpinene (15.5%), *p*-cymene (11.9%), α-terpineol (5.0%), α-terpinene (3.9%), 1,8-cineole (3.5%), α-pinene (2.9%), and terpinolene (2.8%) [44]. Principal components identified from TREO consisted of fenchone (15%), δ-cadinene (11%), 14-hydroxy-β-caryophyllene (8%), and *tau*-cadinol (7%) [46]. The current study reports the major constituents of MCEO as linalool (19%), geranial (16%), citronellal (14%), neral (14%), and sabinene (12%). Since none of these attractive essential oils have major chemical components in common, it is highly likely that a combination of these oils may result in additive or synergistic attraction of male *C. capitata*, as has been observed with multi-component lures for other agricultural pests [48]. Future research is warranted to evaluate efficacy of various combinations of attractive essential oils for improved detection of *C. capitata* under field conditions.

## 3. Materials and Methods

### 3.1. Plant Material

*Magnolia citrata* leaves were collected from Ha Giang Province, Northwestern Vietnam. The GPS was recorded as 23°03′20″ N 105°05′48″ E, and plant material all collected from a single tree. The plant specimen was identified by Mrs. Tu Bao Ngan. A voucher specimen has been deposited at the herbarium of Vietnam National Museum of Nature (VNMN).

### 3.2. Essential Oil Isolation

The harvested leaves (900 g) were subject to hydrodistillation using a Clevenger apparatus (Thermo Fisher Scientific, Waltham, MA, USA) for 3 h. The slightly yellow oil was dried over anhydrous sodium sulfate to remove any trace of water and stored in sealed glass vials at 4 °C until further analysis. The yield was calculated as 0.38% according to the volume of obtained MCEO and was expressed on a fresh weight basis (*v*/*w*).

### 3.3. GC-MS Analysis

The MCEO was diluted in methylene chloride in a 1:1000 ratio for analysis by GC-MS using an Agilent 5975B (Agilent Technologies, Santa Clara, CA, USA) system equipped with a DB5-MS column (Agilent Technologies, Santa Clara, CA, USA). The carrier gas was helium, with a flow rate of 1.3 mL min^−1^. GC oven temperature was kept at 45 °C for 1 min and increased to 94 °C at a rate of 4 °C min^−1^, then increased to 180 °C at a rate of 2 °C min^−1^. The PTV injector temperature was 200 °C. Mass spectra were recorded at 70 eV. Mass range was *m*/*z* 35 to 450, ion source temperature was 230 °C, and the scan rate was 2.8 s^−1^.

#### Identification of Components

Using MassHunter software (B.07.02, Agilent Technologies, Santa Clara, CA, USA), the percentages of each component were calculated based on total ion current without standardization, by means of the chromatographic peak area normalization method. The MCEO components were identified by comparison of their relative indices to C_8_–C_22_ *n*-alkanes that were calculated on a nonpolar DB-5MS column according to the method of van den Dool and Kratz [49], authenticated samples ((*Z*)-3-hexen-1-ol (Cas # 928-96-1, Sigma-Aldrich, St. Louis, MO, USA), α-pinene (Cas # 80-56-8, Sigma-Aldrich, St. Louis, MO, USA), sabinene (Cas # 3387-41-5, Sigma-Aldrich, St. Louis, MO, USA), β-pinene (Cas # 18172-67-3, Sigma-Aldrich, St. Louis, MO, USA), 6-methyl-5-hepten-2-one (Cas # 110-93-0, Sigma-Aldrich, St. Louis, MO, USA), myrcene (Cas # 123-35-3, Sigma-Aldrich, St. Louis, MO, USA), *p*-cymene (Cas # 99-87-6, Sigma-Aldrich, St. Louis, MO, USA), limonene (Cas # 5989-27-5, Florida Chem. Co., Winter Haven, FL, USA), 1,8-cineole (Cas # 470-82-6, Fluka Chemical Co., Buchs, SG, Switzerland), mixture of (*Z*)- and (*E*)-β-ocimene (Cas # 13877-91-3, Sigma-Aldrich, St. Louis, MO, USA), γ-terpinene (Cas # 99-85-4, Sigma-Aldrich, St. Louis, MO, USA), mixture of *cis*- and *trans*-linalool oxide (Cas # 60047-17-8, Sigma-Aldrich, St. Louis, MO, USA), linalool (Cas # 78-70-6, Sigma-Aldrich, St. Louis, MO, USA), mixture of *cis*- and *trans*-*p*-menth-2-en-1-ol (product # 3361 Synergy Semiochemicals Corp., Burnaby, BC, Canada), terpinen-4-ol (Cas # 20126-76-5, Sigma-Aldrich, St. Louis, MO, USA), α-terpineol (Cas # 10482-56-1, Sigma-Aldrich, St. Louis, MO, USA), piperitone (Cas # 89-81-6, Tokyo Chemical Industry Co., Ltd., Tokyo, Japan), β-caryophyllene (Cas # Cas # 87-44-5, Sigma-Aldrich, St. Louis, MO, USA), α-humulene (Cas # 6753-98-6, Sigma-Aldrich, St. Louis, MO, USA), alloaromadendrene (Cas # 25246-27-9, Sigma-Aldrich, St. Louis, MO, USA), and caryophyllene oxide (Cas # 1139-30-6, Sigma-Aldrich, St. Louis, MO, USA)], and by correlating mass spectra to databases of Adams library [33], NIST 17 [34], Wiley 11 [35], MassFinder terpenoids library [36], Flavors and Fragrances of Natural and Synthetic Compounds 3 (FFNSC 3) [37], and our own library “SHRS Essential Oil Constituents-DB5-MS” [50].

### 3.4. Biologicaal Activities

#### 3.4.1. Mosquito Assays

Two strains of *Aedes aegypti* were used for bioassays: Orlando 1952 strain (ORL) and Puerto Rico strain (PR). Mosquitoes were reared following standard procedures at the USDA-ARS Center for Medical, Agricultural, and Veterinary Entomology (CMAVE) in Gainesville, FL, USA [51,52]. Since pyrethroid resistance is common in *Ae. aegypti*, we included adulticidal assays using the strongly resistant PR strain [53]. Newly emerged mosquitoes were maintained on 10% sugar water and kept in laboratory cages at an ambient temperature of 28 ± 1 °C and RH of 35–60%.

#### 3.4.2. Larvicidal Bioassay

Larvicidal activity was assayed against 1st instar *Ae. aegypti* (ORL strain) as described previously [51,52]. Briefly, the MCEO was diluted in dimethyl sulfoxide (DMSO, Cas# 67-68-5, Sigma-Aldrich Inc., St. Louis, MO, USA) to make 100 μg/μL and assayed in four different concentrations (1.0, 0.5, 0.25, and 0.1 μg/μL) in a final volume of 200 μL of larval rearing media into 96-well plates. Mortality was recorded after 24 h of exposure. Larva were considered dead if they did not move during observation or after swirling the contents of the well. Three replicates were conducted on consecutive days. Technical-grade permethrin (Chem Service, West Chester, PA, USA) was used as a positive control at 31 pg/μL, and DMSO was used as the negative control in all assays.

#### 3.4.3. Adulticidal Bioassay

Females of *Ae. aegypti* (ORL and PR strains), 3–6 days post-emergence, were used in toxicity assays as described earlier [42,51,52,53]. Mosquitoes were briefly anesthetized at 4 °C and then sorted by sex. Females were used for all replicate tests. Compounds were diluted from a 100 μg/μL stock in DMSO into a 10 μg/μL working stock in acetone. A 0.5 μL droplet of each working stock was applied to individual mosquitoes (15–20 per dose). A negative control of acetone (Cas # 67-64-1, Sigma-Aldrich Inc., St. Louis, MO, USA) and positive control of permethrin (0.69 ng/μL) (Chem Service, West Chester, PA, USA) near the LD_50_ of the ORL1952 strain was included in each assay. Mortality was scored at 24 h after application, and data were corrected for background mortality with Abbott’s correction if mortality in controls was between 10–20%. Three replicates of the standard adult topical screening bioassay were conducted with 20 female mosquitoes at each dose.

#### 3.4.4. Fruit Fly Assay

Experimental insects consisted of sterile male *C. capitata* obtained from the Programa Moscamed mass rearing facility in El Pino, Guatemala. Flies were irradiated as pupae two days prior to eclosion with 95 Gy of gamma radiation from a ^60^Co source. Irradiated pupae were shipped initially to the USDA-APHIS Medfly Project in Sarasota, FL, USA, and then to the USDA-ARS Subtropical Horticulture Research Station (SHRS) in Miami, FL, USA. Flies were reared using established protocols [44,47] and used in bioassays when sexually mature (5–10 days old).

#### Short-Range Attraction Bioassay

Laboratory bioassays were conducted using methods similar to those reported previously [45,46,47]. Tests were performed using small screen cages (20.3 × 20.3 × 20.3 cm) into which 50 flies were introduced 1 h before the start of an experiment. Experiments were initiated by introducing a Petri dish (53 mm diameter 12 mm height) containing essential oil samples applied to a filter paper disk (Whatman #1, 3.5 cm diameter). Each sample consisted of 10 µL of a 10% dilution in acetone. To determine relative attraction to MCEO, bioassays were performed with several control essential oils for comparison. Treatments consisted of MCEO, TTO (tea tree oil, derived from *Melaleuca alternifolia* (Maiden and Betche) Cheel., [44], TREO (*Tetradenia riparia* (Hochst.) Codd essential oil, [46]), BTEO (blue tansy essential oil, derived from *Tanacetum annuum* L., [47]), and MGEO (mastic gum essential oil, derived from *Pistacia lentiscus* L. var. *chia*, [45]). TTO and TREO are known to be strong attractants of male *C. capitata* and BTEO and MGEO are mild attractants. Tests were run for 30 min; results were recorded initially as the number of flies within a Petri dish, which was then converted to percentage of flies attracted. Tests were replicated five times, and the results were analyzed by ANOVA followed by mean separation with Tukey test (*p* < 0.05) (Systat Software Inc.: San Jose, CA, USA) [54]).

## 4. Conclusions

Plant extracts and oils provide complementary ways of managing pests and diseases especially in tropical and subtropical regions. Due to the adverse effects of synthetic insecticides on the environment and human health, it is necessary to develop alternative control strategies to reduce the use of insecticides. In the present study, for the first time, we investigated the leaf essential oil of *Magnolia citrata* from Vietnam and its biological activity against two major insect pests of agricultural, medical and veterinary importance. Although MCEO exhibited weak toxicity against 1st instar larvae and adult female *Ae. aegypti*, the oil showed a moderately strong attraction to sterile male medfly *C. capitata* as compared to a known strong attractant for medfly, tea tree oil (*Melaleuca alternifolia*). The oxygenated monoterpenes dominated in the MCEO and aliphatic aldehydes represented over three-quarters of the total composition. The presence of aliphatic aldehydes in high concentration might reduce the captures of medflies. However, additional experiments may be required to determine whether aliphatic compounds are responsible for the moderate activity and whether the combination of MCEO with other plant essential oils, or essential oil components, can enhance the effectiveness of sterile male of *C. capitata*. In addition, combination blends should be tested in the field to ensure their efficacy.

## Data Availability

Data is contained within the article.

## Sample Availability

MCEO is available from N.A.L.-D.
